# Prosecution for Malpractice

**Published:** 1846-11

**Authors:** 


*Prosecution for Malpractice.*—We copy the following from the Southern Medical and Surgical Journal.

"The Western Journal of Medicine and Surgery (August, 1846,) contains the details of "A case of Aneurism of the Brachial Artery, on which an action was brought for mal-practice, with recovery of damages." The artery was punctured by Dr. Geohagan in performing the operation of venesection, which caused a false aneurism, requiring the ligation of the brachial artery. An action for damages having been brought in one of the Circuit Courts of Kentucky in 1844, the plaintiff recovered damages to the amount of $275."

"*Another prosecution for Malpractice.*—An action for damages in the Circuit Court of Kalamazoo, Michigan, against Dr. Nathan M. Thomas, resulted in a verdict of $300 in favor of the plaintiff. A new trial had been granted, but at the same time it was taken out of Court, and referred to Dr. Z. Pitcher, of Detroit, Michigan, and Dr. Dan'l Brainard, of Chicago, Ill., as arbitrators. We copy the following award from the Illinois and Indiana Medical and Surgical Journal.

"The undersigned, to whom was submitted the matter in difference between John Beals and Nathan M. Thomas, having carefully examined the injured limb of Daphne Beals, and heard the testimony adduced in relation to the injury and its treatment, and the arguments of council in the case, respectfully report to the Hon. Chief Justice of the State of Michigan, presiding over the Circuit Court of Kalamazoo County,

"That they agree in the opinion, that the said injury was a dislocation of the upper end of the radius forwards, not detected at the time of its occurrence, and that the defendant is therefore liable to the imputation of mal-practice, from defective anatomical knowledge.

"Taking into consideration that this dislocation is one of rare occurrence, and has in several cases been found to be incapable of reduction, and that it was not in this instance attended by the usual distinguishing signs, and was obscured by considerable swelling.

"Considering farther, that the study of anatomy essential to the proper treatment of such cases, is by the laws of the State of Michigan a penitentiary offence; together with the fact that the limb is still highly useful, and may, in our opinion, be essentially improved by judicious treatment, we award that the defendant in this case, is not justly liable to any damages, and (if it belongs to the board to decide this question,) we further award that each party shall pay his own costs.

ZINA PITCHER.
"Kalamazoo, June 13, 1846. DANIEL BRAINARD."

